# A Comprehensive Quantitative and Biological Neural Network Optimization Model of Sports Industry Structure Based on Knowledge Mapping

**DOI:** 10.1155/2022/1158509

**Published:** 2022-08-29

**Authors:** Zhaohong Wang, Yang Gao, Wenge Li

**Affiliations:** ^1^College of Physical Education and Sports, Beijing Normal University, Haidian District, Beijing 100875, China; ^2^Kehua School, Nanshan Foreign Language School, Shenzhen, Guangzhou 518057, China

## Abstract

In this paper, a comprehensive quantitative and biological neural network optimization model of sports industry structure is thoroughly studied and analyzed using knowledge graphs. To address the problems of poor performance interpretability deficiency of knowledge graph-based recommendation methods in the face of relational sparse graphs, a pretraining-based implicit characterization algorithm strategy is proposed for the recall stage, which can solve the problems of difficulty in going online and high delay in the recall stage of the recommendation system while improving the accuracy, and not only this can be applied in the recall stage, but also the sorting and postsorting modules can be used as features. To study the relationship between signaling activity and energy metabolism of pyramidal neurons, an empirical model of the synaptic vesicle cycle is proposed to simulate the synaptic transmission process, the role played by energy metabolism in synaptic transmission is studied from the perspective of feedback control, and the quantitative relationship between neuronal pulse discharge frequency, energy consumption, and information quantity in dendritic integration is analyzed using the cable theory and atrial chamber model. It was found that, when 0 ≤ *ε* ≤ 0.6, the chaotic region shrinks and eventually disappears with the increase of the memory factor *ε*; however, when 0.6 ≤ *ε* ≤ 1 is used, chaos is recreated and the chaotic area gradually increases with the increase of the memory factor *ε*. This paper conducts comparative experiments on data sets in the recommendation domain and verifies that the proposed model and the feature intersection module can effectively perform feature interaction between items and entities, thus enhancing the recommendation effect.

## 1. Introduction

The existing studies often have poor recommendation performance when facing graphs with sparse relationships and lack certain interpretability. Meanwhile, as the link between knowledge utilization and creation, the academic field is flooded with a large amount of knowledge and recommendation systems need to be introduced to solve the information overload problem. Increased platforms are providing services for human beings through the Internet, almost all over the clothing, food, and housing of people's lives [1]. Since the birth of the Internet, the development of related technologies has been advancing all the time, and people can access more information by going online to fully enjoy the convenience of the Internet. Internet technology has brought unprecedented changes to all aspects of people's lives [2]. The way people buy goods has changed from offline to online without leaving home, the way they taste food has changed from dining out to delivery, and the sales method of each merchant has changed from local sales in physical stores to online sales in multiple ways; but, at the same time, people are also in an era of hyperinformation, and it is difficult for them to choose what they want from the huge amount of information. In recent years, many researchers have fused recommendation systems with relevant auxiliary information to improve the effectiveness of recommendations, and knowledge graphs are one of the most popular types of structural information. Knowledge graphs cover different domains, such as academics, films and television, music, etc., which makes it very convenient to use domain knowledge graphs to assist recommendation systems.

In recent years, with the rapid development of neuroscience, artificial intelligence, and other technologies, the great potential of brain science has been highly valued, and the China Brain Project, which has been in the making for many years, has been launched. The development of brain science not only contributes to the prevention and treatment of brain diseases but also promotes the development of brain-like intelligence [3]. In the field of brain science, the simulation modeling of biological neurons and their networks occupies an important position and is a popular research direction in the field of brain science. On the one hand, simulation modeling of biological neurons and their networks helps to understand the working principle of the nervous system from a global perspective and to recognize the role played by various neural mechanisms in the operation of the brain; specifically, due to the limitations of experimental conditions and observation techniques, the physiological activities of a small number of neurons can only be observed simultaneously at mesoscopic or microscopic scales, and it is difficult to observe the physiological activities of large-scale biological neural networks at the same time. In addition, simulation modeling can study the effect of certain factors on neural network activity by adjusting parameters, whereas biological experiments require the creation of many control and experimental groups to accomplish this task, which is more flexible and convenient [4]. On the other hand, simulation modeling of biological neurons and their networks helps artificial intelligence technologies break through bottlenecks and move toward strong artificial intelligence. The goal of artificial intelligence technology is to produce machines that can simulate human thought processes and intelligent behaviors, or even surpass human intelligence. A prerequisite for achieving this goal is to master how the brain works and then simulate the brain's cognitive functions. The success of current AI technologies is largely due to mathematical optimization and is far from the workings of the real brain. Although AI technologies have been widely used in various fields, their level of intelligence is still far from human intelligence. The focus of simulation modeling of biological neurons and their networks is to explore and learn from the working principles of the brain rather than mathematical optimization. Therefore, simulation modeling of biological neurons and their networks can help current artificial intelligence break the shackles and improve the level of intelligence.

With the steady and rapid development of the economic environment, the sports industry is receiving higher and higher attention, both in terms of scale and quality, which is most directly reflected in the number of relevant employees and added value. The value-added created reached 325.59 billion yuan, accounting for 0.62% of the total national GDP in that year, maintaining an increased rate of more than 10% for ten consecutive years, which is significantly higher than the growth rate of the overall national GDP. The sports industry has grown into a new hotspot of economic growth, especially in some developed eastern provinces and cities, the scale of the industry and its ability to absorb employment has been no different from the level of medium developed countries in Europe and the United States at the end of the last century [5]. The analysis and research of industrial development based on the theory of industrial structure is one of the research hotspots of global scholars in recent years. At the same time, with the development of society, the sports industry is facing new development opportunities and challenges, among which the industrial structure is one of the bottlenecks. Objectively speaking, compared with Western countries, our research in this field has started relatively late and we have not systematically evaluated and analyzed the current situation of sports industry structure development from a macro perspective [6]. With this background, the author firstly composes the existing theoretical concepts related to the optimization of sports industry structure, then makes a more comprehensive and objective evaluation of them, lists the objective problems based on the in-depth understanding of the development process and change trend of the sports industry, and finally proposes feasible strategies for the optimization of sports industry structure from a practical perspective. The final proposal is a feasible strategy for optimizing the structure of the sports industry from a practical perspective. On the one hand, it provides the necessary theoretical basis for the government to formulate the development plan for the sports industry, and on the other hand, it makes an important contribution to the realization of the dream of a “strong sports nation.”

## 2. Related Works

With the development of knowledge graphs, it has become much more than a simple semantic network, but a technical system containing entities, attributes, concepts, and various rich semantic relationships. Compared with traditional semantic networks, knowledge graphs have a huge scale, rich semantic information, refined quality, and a more friendly structure [7]. The knowledge graph is a large-scale semantic network that generates new knowledge by acquiring and integrating information into a knowledge base and then reasoning about many entities, attributes, and semantic information between entities. As a technical system, knowledge graphs have a wide range of applications in cognitive intelligence fields such as data analysis, search engines, recommendation systems, and human-computer interaction, and corresponding research projects related to knowledge graphs have been conducted in domestic commercial fields as well as academic fields. The knowledge graph is regarded as a third-order tensor, and low-dimensional entity embedding is used, and low-dimensional entity embedding and relationship embedding are used to reduce the third-order tensor. Neural network-based and graph neural network-based approaches, such as R-GCN, fuse entities with information about other entities associated with them through graph convolutional neural networks to obtain a semantic representation of the target entity [8]. Embedding-based approaches use the translational distance model to implement entity embedding, obtain the corresponding entity representation through embedding, and use the entity representation to achieve recommendation. Yang et al. proposed an embedding-based collaborative knowledge recommendation approach (CKE) in 2016, which extracts structured content, textual content, and visual content from the knowledge graph and uses the TransR model, noise reduction autoencoders, and convolutional autoencoders to embed these three types of content representations [9]. Qingwen et al. in 2019 proposed a multitask feature learning recommendation model (MKR), which designs a cross-compression unit that enables knowledge sharing between the embedding part and the recommendation part and implements recommendations accordingly [10]. The fusion-based approach takes the entity relationships of the knowledge graph and optimizes the item or user vector in the recommendation algorithm by convolutional neural networks, graph neural networks, etc. Baskonus et al. simulated the propagation process of user interests on the knowledge graph and portrayed the user preferences in more detail using the relational information within the knowledge graph [11]. The recommendation methods based on knowledge graphs have different advantages and disadvantages, but they generally have the problem of unsatisfactory recommendation effect when dealing with knowledge graphs of sparse relationships.

As a sunrise industry supporting the rapid development of the national economy, the internal structure of the sports industry is also inextricably linked. It is especially important to follow the rules of economic development and sports industry development to have a comprehensive understanding and grasp of its industrial structure. As an indispensable and important part of the sports industry, the importance of the structure of the sports industry has become increasingly prominent. After years of development and precipitation, the academic research results on the current situation of sports industry structure are richer, with the foothold of research rising from the perspective of the whole country, as well as combining the actual situation of field investigation in provinces, cities, and autonomous regions, and comprehensive analysis of the sports industry from the overall perspective, as well as a study on its composition and development trend from a micro perspective. According to Gupta and Chandra, the structure of the sports industry refers to the technical and economic links and quantitative proportional relationships among the production sectors in the industry [12]. On the one hand, it can comprehensively reflect the interdependence and mutual constraints in production technology among the production sectors of sports goods and sports services, and on the other hand, it can reflect the current allocation of various economic resources in each sector and the specific distribution of the total output value of the sports industry in each sector. There are still some scholars who define the structure of the sports industry from different disciplinary perspectives. Novak et al., from the perspective of sports consumption, believe that the structure of the sports industry is the arrangement, elements, and configurations of various sectors in the context of the sports industry, which needs to optimize the allocation of sports resources in a market-oriented way to meet the objective needs of the public as much as possible [13]. From a macro perspective, some scholars believe that the structure of the sports industry is “the linkages between sports industries and the contacts they form.” It is known that there are also structural links in the internal structure of the sports industry. It should be noted that, when studying the internal links of the structure of the sports industry, it should be assumed that the external conditions remain unchanged and the ratio of inputs and outputs within the sports industry should be considered as the focus of research. Ren et al. analyzed the contradiction between supply and demand in the structure of China's sports industry and points out that the core part of China's sports industry accounts for less than 20% and is still in the primary development stage, and the main problem facing the supply and demand of China's sports industry structure is the insufficient supply of sports products and the ineffective supply of some sports goods [14].

Neurons are the structural units of the nervous system, which is made up of many interconnected neurons. Neural behavior is not determined by individual neurons but is the result of the interaction of neuronal populations. Therefore, studying the topological properties and structural evolution of the nervous system, clarifying how neurons establish synaptic connections, how synaptic connections change, and other fundamental issues are necessary prerequisites for mastering the operating mechanism of the brain. In the research process, biological neural network simulation modeling is an indispensable technical tool. In terms of identification methods, two main types of methods are currently used for the identification of biological neural network systems [15]. The first is to use adaptive state observer for identification. By constructing a following system with the same structure and unknown parameters to be identified, and based on Liapunov stability theory, a parameter adaptive adjustment equation is constructed, so that the parameters to be identified converge to the actual parameter value when the time tends to infinity.The second is to identify modern optimization algorithms based on strong optimization algorithms. The identification problem of biological neural network system is transformed into an optimization problem by constructing an appropriate objective function. Modern optimization algorithms are used to find the best solution, so as to obtain a more satisfactory solution through continuous iterative search, that is, to identify unknown parameters or unknown topologies. The simulation modeling of biological neural networks refers to the creation of biologically interpretable network models that can elaborate certain properties or functions of the nervous system based on the current knowledge of the nervous system, such as the study of synaptic plasticity, topological properties, and structural evolution of the nervous system. Jinfeng and Bo confirmed the validity of Hebb's theory [16]. Chen et al. investigated the molecular mechanisms underlying LTP and LTD and showed that most glutamatergic synapses exhibit LTP and LTD concerning the activation of NMDA receptors on the postsynaptic membrane and calcium ion concentration [17].

## 3. Construction of a Comprehensive Quantitative and Biological Neural Network Optimization Model of Sports Industry Structure Based on Knowledge Mapping

### 3.1. Knowledge Graph Model Design

Knowledge graph construction technology is a complex technical system that requires knowledge extraction, knowledge fusion, knowledge processing, and other techniques to support it. Starting from heterogeneous data and ending with the formation of a knowledge graph, many key technologies are involved in the middle, including knowledge acquisition, processing, and structured representation [18]. Knowledge extraction refers to extracting valuable knowledge from multiple heterogeneous information sources. Knowledge fusion is to resolve the ambiguity of knowledge by using related technologies and to form a standard knowledge base by fusing multiple knowledge bases. Knowledge processing is the process of inferential representation of knowledge, which determines the final quality of the knowledge graph. The stability of the single-task model is insufficient. ESMM considers everything from exposure to click to conversion.

(1) $$\begin{array}{c} I=\left\{u,U_{s},S\right\}. \end{array}$$

**Figure 1:**
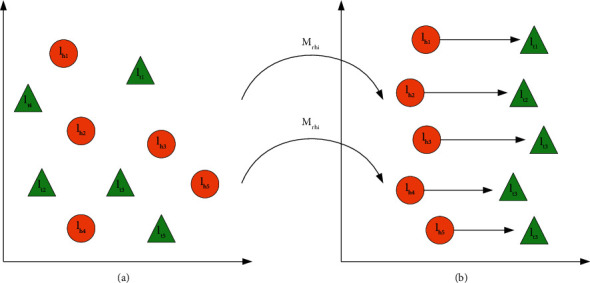
TransR mapping relationship diagram: (a) entity space and (b) relative space of *r*.

Knowledge fusion is mainly used to solve the problem of the heterogeneous knowledge graph, and it is mainly realized through entity alignment. According to the scholar's information obtained from unstructured data and semistructured data, it is necessary to clean them first, then construct triples according to the cleaned data, and finally align the two groups of triples data through entity alignment. The data obtained from unstructured data are extracted by using a relatively mature algorithm, and the accuracy of entity recognition is also high. Therefore, the main object of data cleaning is the data obtained from semistructured data. As shown in Figure 1, it is indicated that TransR uses the relation-specific matrix. Maps*M*^*r*^*h*and*t*to subspace*R*^*k*^as follows:

(2) $$\begin{array}{c} V_{h}^{r}=v_{h}M^{r},v_{t}M^{r}. \end{array}$$

**Figure 2:**
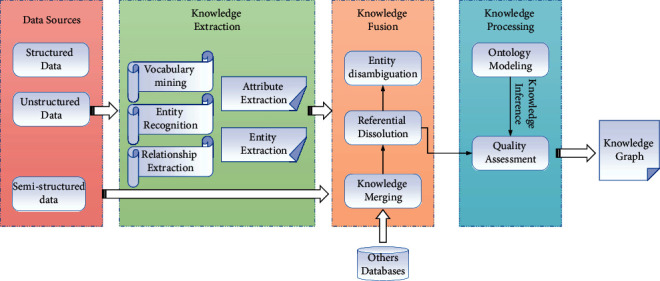
Flow chart of knowledge graph construction.

The EL is debugged through predefined entities of user reviews and knotted food for a specific product, using DBpedia as the reference set . First, the NER system was used to distinguish between structured and unstructured texts and filter the DBpedia for irrelevant entities. Of course, many publicly available entity identification tools can also be used. The scoring functions of the embedded technology reference are classified into the translation distance model based on the distance scoring function and the semantic matching model based on the similarity scoring function [19]. The process of knowledge graph construction is shown in Figure 2.

For the semistructured data of Scholars.com, certain extraction rules need to be designed to extract the data from the Web pages automatically; while for the unstructured data, the corresponding algorithm needs to be used to extract them and the BERT-BiLSTM-CRF model with excellent stability and accuracy in recent years is chosen here. After obtaining the two data, we need to clean the data, then construct the triad data. After the knowledge fusion is completed, certain id mapping relationships are set for the triad and the entity. Finally, the entities and relationships are stored in Neo4j in bulk using the import method. At this point, the nodes, relationships, and the knowledge graph of scholars formed by them can be viewed in the graph database. Due to the differences in the structured of semistructured scholar data, it is obvious that extraction using algorithms is not easy to implement. Therefore, it is necessary to design the corresponding entity extraction rules by hand and use the rules to extract the semistructured data from the Web pages and transform them into structured scholarly data.

### 3.2. Comprehensive Quantitative and Biological Neural Network Optimization Model Construction for Sports Industry Structure

Considering the complexity of the sports industry, to fully analyze the structure of the sports industry, we should recognize the links between the sports industry and other industries, as well as the links between the elements of interest within the sports industry. On the one hand, the direct structural size of different elements of the sports industry in terms of the scale of “input-output” specifically expresses the characteristics of the structural volume of the sports industry; and on the other hand, the arrangement of different elements in the industrial content has different structural characteristics. The structural form of the sports industry is specifically expressed in the different levels of the industrial structure [20]. Considering the problem of the extensiveness and complexity of different industrial links, we should analyze and study the structure of the sports industry from multiple levels and perspectives in the research process. Accordingly, we can also analyze the structural form of the sports industry from the following aspects: industry structure, hierarchical structure, organizational structure, ownership structure, market structure, regional structure, etc. 

**Figure 3:**
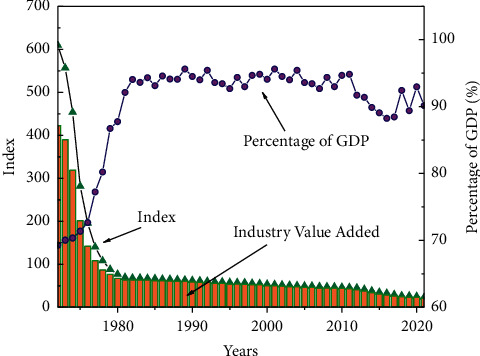
Value added of the national sports industry and proportion of GDP.

The sports service industry includes other sports industries except for sports goods and manufacturing and sports venue facilities construction, including a total of seven categories; the proportion of each category is relatively small, and the quality of supply and service efficiency of the sports service industry are not high enough. Therefore, after more than a decade of growth and rapid development, China's sports industry is growing and maturing, with rapid growth in scale, continuous improvement of the industrial system, and increasing enrichment of industrial categories. China's sports industry is growing rapidly and significantly higher than the growth rate of the macro economy in the same period, with a total scale of more than 1 trillion yuan, and the proportion of China's GDP is also increasing, accounting for about 1.14% of GDP in 2019. As shown in Figure 3, it reflects the value added of China's sports industry and its share in GDP in recent years.

Based on the continuous optimization and adjustment of the sports industry structure, we have transformed the quantitative and economic-technical links between sports industries from the previous uncoordinated state to the current coordinated state. In other words, the restructuring of the sports industry involves two different aspects: market adjustment and government regulation. Among them, the market adjustment belongs to the category of natural adjustment, while government regulation belongs to the category of artificial adjustment. In the current market economy, we mainly do the optimal allocation of market resources among sports industries under the role of market value law; at the same time, from the government level, the government mainly adjusts industrial policies and other macro-control methods and then can provide institutional support for the healthy development of sports industry from the institutional level and effectively promote the rationalization of sports industry development level. This means that changes in cell membrane potential do not occur synchronously, and local membrane potential changes can cause a chain reaction across the cell membrane.

**Figure 4:**
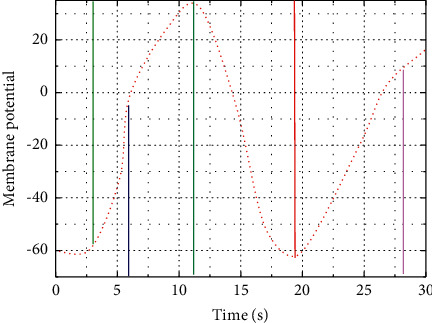
Mechanism of action potential generation.

This ionic osmotic pressure together with the unique ionic permeability of the cell membrane leads to the generation of the cell membrane potential, which in turn indirectly changes the ionic permeability of the cell membrane and the ionic osmotic pressure inside and outside the membrane by affecting the opening and closing state of voltage-gated ion channels [21]. The cell membrane potential can be divided into resting and action potentials, as shown in Figure 4. When a biological neuron is not stimulated, the ionic osmotic pressure inside and outside the cell membrane, the cell membrane potential, and the ionic permeability of the cell membrane are in equilibrium, and the cell membrane potential is in a stable state at this moment is called the resting potential. It is found that the potassium channels in the cell membrane are open and the sodium channels are closed, the high potassium ion osmolarity inside the cell membrane and the positive voltage outside the cell membrane cancel each other out, and the cell membrane potential is stable at about 65 mV.

Biological neurons have a complex morphological structure, and their dendrites, cell bodies, and axons may be distributed far apart in space, and stimulation of the cell membrane often occurs in local areas, which means that changes in the cell membrane potential do not occur synchronously, and local membrane potential changes can cause a chain reaction in the cell membrane. For example, a stimulus signal causes a change in membrane potential in a local region of the dendrites of a biological neuron, which in turn disrupts the equilibrium in the neighboring region, which in turn causes a change in membrane potential in the neighboring region. Because of the spatial differences in the morphological structure of biological neurons and the ion permeability of cell membranes, and because biological neurons usually receive many stimulus signals simultaneously, these stimulus signals are constantly scaled, filtered, fused, and produce other complex nonlinear changes when they are transmitted within biological neurons. Static neurons are neurons whose state does not change over time.

(3) $$\begin{array}{c} f\left(x\right)=\sum_{i=1}w_{ij}+f\left(x_{\text{i}}\right)-\theta_{j}, \end{array}$$

where *x*_*i*_(*i* = 1,2,…, *m*) is the incoming signal of other neurons, *θ*_*j*_ is the bias of neuron, *w*_*ij*_ is the connection weight of the *i*-th neuron and *j*-th neuron, *f* is the activation function, the sigmoid function is one of the commonly used activation functions, and *f*(*x*) is the output of the neuron. The structure of the static neuron model is very simple, the weighted summation of the input stimulus simulates the information integration function of the cell body of the biological neuron, the bias *θ*_*j*_ simulates the threshold potential of the biological neuron, and the nonlinear activation function simulates the nonlinear properties of the biological neuron.

## 4. Analysis of Results

### 4.1. Knowledge Graph Model Results

**Figure 5:**
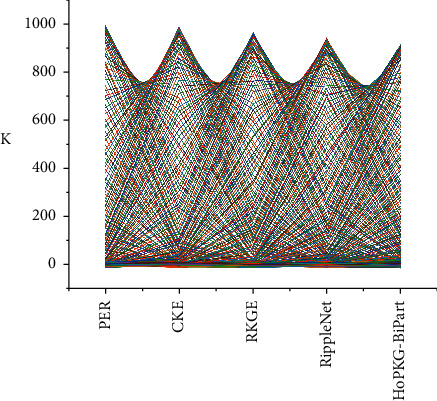
Performance comparison results of the model.

Compared with traditional semantic network, knowledge graphs have the characteristics of huge scale, rich semantic information, excellent quality, and more friendly structure. The core of the advanced industrial structure is the innovative use of high-level production factors such as science and technology and the optimization of the transformation capacity of the industrial structure. The process of the heightening of the sports industry structure is due to the different degrees of productivity improvement in various sports industries or the different income elasticity of demand for sports products, resulting in the differentiation of the development speed of various sports industries and changes in their dominant positions, resulting in a new adjustment of the sports industry structure. In addition, the best entity embedding dimension will be selected {32,64,128,256,512}*d*; the best depth of the propagation layer will be selected {1,2,3,4,5}*l*. Each set of experiments will be repeated 10 times, and the average of the best results will be taken. As shown in Figure 5, HoPKG also performs best in Rec@K and Pre@K compared with other methods. The reason PER and MCRec do not perform as good as other methods is that they require manual construction of meta-paths, which will lead to some uncertainty factors. Therefore, the performance is more general when the data set is sparse. In addition, different aggregation methods can produce different results. The dual aggregation method BiPart proposed in this paper has some improvement over GraphSAGE in both AUC and F1 since the HoPKG model combines entity representations aggregated in two different ways and can propagate entity information more effectively.

MF can only characterize features and end targets and has poor performance in constructing contexts. nFM treats user-goods interactions as features and models them with linear neural networks and nonlinear multilayer perceptrons, thus enabling better predictions than FM [22]. However, due to single-task modeling, there are many possibilities for the distribution in the links, resulting in sparse samples of depth events and large fluctuations in the distribution of depth events when performing single-task modeling of deep links. The stability of the single-task model is not sufficient. eSMM considers the transition from bursts to clicks and back again. Modeling is only applicable to the e-commerce case. The set-up assumption is *ctcvr*=*ctr* × *cvr*, but this may not be the case in real situations. The results suggest that appropriate neural networks can improve feature learning in the user interaction information. The performance of ESMMV2 is mainly due to its deeper modeling links and the addition of more mid-question states to assist in modeling. Again, the modeling still depends on the strong correlation between the deep and shallow tasks that complete the modeling. The changing correlations of user intent are not fixed, and the performance in terms of metrics is not satisfactory.

### 4.2. Comprehensive Quantitative and Biological Neural Network Optimization Model Performance Test of Sports Industry Structure

The sectoral analysis chart is a system of coordinates with the deviation component *PD*_*ij*_ on the horizontal axis and the share component *N*_*ij*_ on the vertical axis, in which the scatter points representing each industry sector are marked. The greater the value, the greater the ability of the region's industrial sector to contribute to the total volume of the sports industry. Nissl bodies are where proteins are synthesized and are involved in the synthesis of the neurotransmitter acetylcholine. The essence of neurofibrils is the aggregation of neurofilaments and nerve microtubules in neurons when they are fixed, and their functions are related to material transport and axonal growth.

Among the eight sectors of the sports industry, the competitive weight of “sports goods, clothing, footwear, and hat manufacturing,” “sports construction activities,” “sports goods, clothing, footwear, and hat sales,” “sports stadium management activities,” and “sports intermediary activities” is greater than zero, indicating that the growth rate of these five sectors is greater than the average growth rate of the national sports industry. In particular, the growth rate of “sporting goods, clothing, footwear, and hat manufacturing” and “sports construction activities” is higher than the average growth rate of the national sports industry; the competitive components of the other three sectors are less than 0, indicating that their growth rates are smaller than the average growth rate of the national sports industry [23]. The growth rate of the other three sectors is less than 0, indicating that their growth rate is smaller than the national average growth rate of the sports industry. When the competition component >0, it means that the growth rate is greater than the average growth rate of the national sports industry. When the competition component is less than 0, it means that its growth rate is less than the average growth rate of the national sports industry.

**Figure 6:**
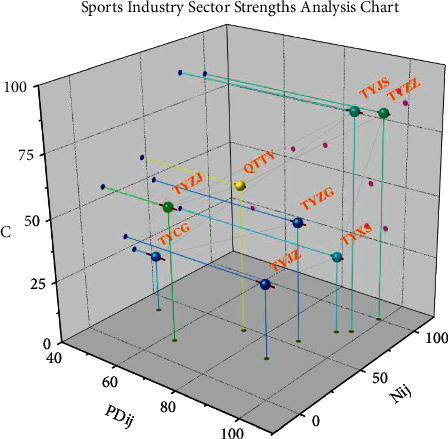
Analysis of the advantages of the sports industry sector.

As shown in Figure 6, from the deviation component, the values of “sales of sports goods, apparel, footwear, and hats,” “sports construction activities,” and “manufacturing of sports goods, apparel, footwear, and hats” are larger, which means that these three sectors have more obvious growth rates. This indicates that these three sectors have more obvious sectoral advantages. In terms of share, “sporting goods, apparel, footwear, and hat manufacturing” has the largest contribution to the total sports industry.

Knowledge subgraphs were constructed for the experiments using DBpedia, and all models were executed in Python. For each item, entities were extracted from their reviews and extended to 3-hop relevant entities. 25% were used as a test set, 25% as a validation set, and the remaining 50% of the original data were used as a training set to obtain average results for five random groupings. The Adam optimizer was used for all models. The learning speed was set to 0.001, and the embedded item dimension and the users and entities were fixed at 128. To be fair, the baseline was also set to the same size. BatchNorm was used to achieve faster and more stable training; research the topological structure characteristics and structural evolution law of the nervous system; and clarify the basic issues such as how neurons establish synaptic connections and how synaptic connections change.

**Figure 7:**
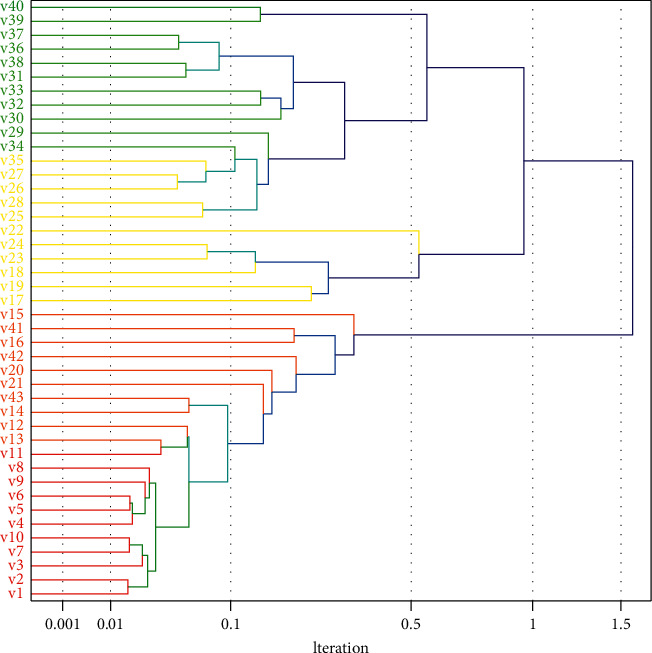
Topology identification results.

As shown in Figure 7, there are errors in both parameters and topology at the beginning, and the errors decrease gradually as the iterations proceed. At the end of the iteration, there is a small error in the parameter identification, while the topology identification error is 0, indicating that the topology is correctly identified. The identification result is as follows: *a*_1_=1.32, *a*_2_=1.59, *a*_3_=2.14, *a*_4_=1.64, *a*_5_=2.83. The initial solution is generated randomly. As the number of iterations increases, the parameter identification error and topology identification error decrease gradually. The final topology recognition error is 0, which means that all topologies are correctly recognized, while there is still a small error in parameter recognition. Since the solution space is larger for all neurons together, all topologies are identified, while there is still a small error in parameter identification.

## 5. Conclusion

This paper builds a comprehensive quantitative and biological neural network optimization model of the sports industry structure with the help of knowledge graphs and conducts in-depth research and analysis on the comprehensive quantitative and biological neural network optimization model of the sports industry structure. Based on the constructed knowledge graph and user-scholar interaction history, we investigate more effective and interpretable recommendation methods. First, for the knowledge graph, the TranSparse method is used to embed the corresponding vector representation, and then according to the characteristics of the knowledge graph, an attention mechanism is introduced to calculate the score between the target node and the neighbor nodes. Second, using the idea of high-order propagation, the entity representation is obtained in the knowledge graph in a hierarchical manner, and a dual aggregation method is proposed to aggregate entity information from two aspects, so that a richer entity representation can be obtained. Finally, experiments are carried out on the data set, and the experimental results show that the method proposed in this paper has certain advantages in recommendation effect and interpretability. The connection strength identification of the Hindmarsh–Rose neural network model is carried out using a real-coded genetic algorithm. The network model identification problem is transformed into an optimization problem, and according to the characteristics of multiple optimization variables of the neural network, the implementation process of the genetic algorithm using real coding is described in this paper, and the algorithm is optimized and improved for the high-dimensional identification problem. On this basis, combined with the actual situation, it should be optimized and adjusted to effectively promote the systematic and scientific development of the sports industry structure. In turn, it can promote the transformation of the sports industry structure from a lower level to a higher level. Although some achievements have been obtained in this paper, there are still shortcomings. The current research uses typical values to fix these parameters in synaptic coupling, but more accurate model identification problems need to take all parameters into consideration.

## Data Availability

The data used to support the findings of this study are available from the corresponding author upon request.

## References

[1] Bai L., Zheng K., Wang Z., Liu J. (2022). Service provider portfolio selection for project management using a BP neural network. *Annals of Operations Research*.

[2] Jiang L., Zhang T., Feng Y. (2020). Identifying the critical factors of sustainable manufacturing using the fuzzy DEMATEL method. *Applied Mathematics and Nonlinear Sciences*.

[3] Zhang R. (2022). Analyzing body changes of high-level dance movements through biological image visualization technology by convolutional neural network. *The Journal of Supercomputing*.

[4] Wang Y., Liu Y., Sun Y. (2020). A hybrid intelligence technique based on the Taguchi method for multi-objective process parameter optimization of the 3D additive screen printing of athletic shoes. *Textile Research Journal*.

[5] Kazemi Garajeh M., Blaschke T., Hossein Haghi V., Weng Q., Valizadeh Kamran K., Li Z. (2022). A comparison between sentinel-2 and landsat 8 OLI satellite images for soil salinity distribution mapping using a deep learning convolutional neural network. *Canadian Journal of Remote Sensing*.

[6] Ma L., Sun B. (2020). Machine learning and AI in marketing – c. *International Journal of Research in Marketing*.

[7] Varsha P. S., Akter S., Kumar A. (2021). The impact of artificial intelligence on branding: a bibliometric analysis (1982-2019). *Journal of Global Information Management*.

[8] Memmert D., Perl J. (2009). Game creativity analysis using neural networks. *Journal of Sports Sciences*.

[9] Yang D., Du Y., Yao H., Bao L. (2022). Image semantic segmentation with hierarchical feature fusion based on deep neural network. *Connection Science*.

[10] Qingwen M., Bojie W., Yehong S. (2022). Progresses and perspectives of the resource evaluation related to agri-cultural heritage tourism. *Journal of Resources and Ecology*.

[11] Baskonus H. M., Bulut H., Sulaiman T. A. (2019). New complex hyperbolic structures to the lonngren-wave equation by using sine-gordon expansion method. *Applied Mathematics and Nonlinear Sciences*.

[12] Gupta M. K., Chandra P. (2020). A comprehensive survey of data mining. *International Journal of Information Technology*.

[13] Novak T. P., Hoffman D. L., Yung Y. F. (2000). Measuring the customer experience in online environments: a structural modeling approach. *Marketing Science*.

[14] Ren Y., Cheng T., Zhang Y. (2019). Deep spatio-temporal residual neural networks for road-network-based data modeling. *International Journal of Geographical Information Science*.

[15] Pathak S. D., Day J. M., Nair A., Sawaya W. J., Kristal M. M. (2007). Complexity and adaptivity in supply networks: building supply network theory using a complex adaptive systems perspective∗. *Decision Sciences*.

[16] Jinfeng L., Bo Y. (2021). Design of evaluation system of physical education based on machine learning algorithm and SVM. *Journal of Intelligent and Fuzzy Systems*.

[17] Chen W., Li X., Chen X., Xiong Y. (2021). Research on influence mechanism of running clothing fatigue based on BP neural network. *Journal of Intelligent and Fuzzy Systems*.

[18] Ren Y., Rubaiee S., Ahmed A., Othman A. M., Arora S. K. (2022). Multi-objective optimization design of steel structure building energy consumption simulation based on genetic algorithm. *Nonlinear Engineering*.

[19] Almarashdeh I., Bouzkraoui H., Azouaoui A. (2018). An overview of technology evolution: investigating the factors influencing non-bitcoins users to adopt bitcoins as online payment transaction method. *Journal of Theoretical and Applied Information Technology*.

[20] Zhang W., Wu Q. M. J., Yang Y. (2020). A width-growth model with subnetwork nodes and refinement structure for representation learning and image classification. *IEEE Transactions on Industrial Informatics*.

[21] Arabameri A., Seyed Danesh A., Santosh M. (2022). Flood susceptibility mapping using meta-heuristic algorithms. *Geomatics, Natural Hazards and Risk*.

[22] Mohamed A., Najafabadi M. K., Wah Y. B., Zaman E. A. K., Maskat R (2020). The state of the art and taxonomy of big data analytics: view from new big data framework. *Artificial Intelligence Review*.

[23] Fonseca S. T., Souza T. R., Verhagen E. (2020). Sports injury forecasting and complexity: a synergetic approach. *Sports Medicine*.

